# Chromatin accessibility dynamics and a hierarchical transcriptional regulatory network for shoot apex cold stress in *Eucalyptus grandis*

**DOI:** 10.48130/forres-0026-0011

**Published:** 2026-04-13

**Authors:** Peishan Li, Shasha Zhang, Xinrong Liu, Jiaojiao Li, Lu Li, Wei Wu, Deming Yang, Zhaohua Lu, Juncheng Lin, Liuyin Ma

**Affiliations:** 1 Fujian Provincial Key Laboratory of Haixia Plant Systems Biology, Haixia Institute of Science and Technology, Fujian Agriculture and Forestry University, Fuzhou, Fujian 350002, China; 2 Center for Seedling, Fujian Yongan Forestry (Group) Joint-Stock Co., Ltd, Yongan, Fujian 366000, China; 3 Research Institute of Tropical Forestry, Chinese Academy of Forestry, Guangzhou, Guangdong 510520, China

**Keywords:** Chromatin accessibility, Transcription factor, Shoot apex, Cold stress, *Eucalyptus grandis*

## Abstract

Subtropical perennials lack formal winter dormancy mechanisms, rendering them more vulnerable to cold stress than temperate or boreal trees. While cold-response pathways are well characterized in model plants, the regulatory landscape in woody species remains elusive. Here, we demonstrate that shoot apices of the fast-growing subtropical tree *Eucalyptus grandis* respond to cold stress within 0.5 h through rapid chromatin accessibility remodeling and transcriptomic reprogramming. Time-series ATAC-seq and RNA-seq analyses revealed a hierarchical regulatory architecture over a 24 h period; this architecture is initiated by hormone- and circadian-related genes, followed by metabolic, cell-cycle, and physiological adjustments. Transcriptomic reprogramming was concentrated in cold- and red-light-responsive genes (e.g., *CBF1* and *CBF4*), which were induced as early as 0.5–2 h post-treatment. Functional validation confirmed that four rapid cold-responsive motifs, including a CBF-binding site, exhibited elevated luciferase activity at 4 °C. While over 50% of dynamic chromatin accessibility regions correlated with differential gene expression, chromatin opening did not always coincide with immediate transcriptional activation. Notably, canonical auxin signaling components exhibited sustained induction and synchronized chromatin-expression dynamics. *De novo* motif analysis indicated that constitutively expressed AGL42 and ERF transcription factors in shoot apices may directly activate *CBF4*, initiating a 'development-to-stress' switch via downstream chromatin remodeling. Direct activation of the *CBF4* promoter by two *Eucalyptus grandis* AGL42 orthologs was confirmed, establishing a novel regulatory relationship in the cold response. These findings provide a high-resolution map of how chromatin accessibility integrates transcription factors into a hierarchical regulatory network to modulate cold adaptation in subtropical woody plants.

## Introduction

Being sessile in nature, plants cannot relocate to evade adverse environmental conditions, rendering their survival perpetually challenged by many environmental pressures^[1−3]^. Many annual plants cope with such challenges through an alternation of generations, thereby escaping harsh seasonal shifts. In contrast, perennial trees must directly confront abiotic stresses, including severe cold stress in winter^[4,5]^. While temperate and boreal trees have evolved sophisticated winter dormancy mechanisms to cope with cold stress, tropical and subtropical trees are significantly more vulnerable^[4,6]^. The absence of winter dormancy in these species results in substantial negative impacts on growth and development upon cold exposure^[7]^. Consequently, cold stress stands out as a critical abiotic factor with profound implications for agricultural productivity and forest ecosystems^[8]^. Prolonged exposure to cold stress, including chilling (0−15 °C) or freezing (< 0 °C) temperatures, negatively impacts plant growth and development by inhibiting root growth, damaging leaves and reproductive organs, delaying seed germination, and reducing photosynthesis and respiration, ultimately leading to decreased yields in crops and impairing the growth and survival of woody plants^[9−13]^. Given these impacts, elucidating the transcriptional and epigenetic landscapes of the cold response is essential for enhancing cold tolerance in woody plants.

To mitigate cold-induced damage, plants have evolved intricate signal transduction pathways^[2,8]^. In the canonical ICE1-CBF-COR regulatory pathway, cold stress triggers the activation of the kinase OST1 by repressing its negative regulators, such as PP2C-type phosphatases (EGR2, PP2CG1, and PP2CG2)^[8,14−16]^. Phosphorylated OST1 subsequently activates the transcription factor (TF) ICE1 by releasing ICE1 from the inhibition of HOS1, MPK3, and MPK6^[17−20]^. The activated ICE1 binds to the promoters of *C-repeat binding factors* (*CBFs*) to drive the expression of *Cold-Regulated* (*COR*) genes^[21]^. Moreover, the ICE1-CBF-COR pathway is highly conserved across different plant species^[8]^. Furthermore, the CPK-NLP-COR pathway facilitates a rapid response; cold stress activates the kinase CPK28 through a Ca^2+^-binding-dependent mechanism within seconds, leading to the nuclear translocation of NLP7 and subsequent *COR* induction via CBFs^[22]^. Other regulators, including CAMTAs, particularly CAMTA3 and CAMTA5, regulate the cold-induced expression of *CBF*s in *Arabidopsis* by activating CBF1 under abrupt, rather than gradual temperature drops^[23,24]^. Conversely, the expression of *CBFs* is also negatively regulated by the transcription factors MYB15 in *Arabidopsis,* and bZIP68 in maize, respectively^[25,26]^. Notably, cold signaling integrates with light signaling through PIF3, PHYB, and HY5, and with circadian clock signaling via RVE4/RVE8 and PRRs, to regulate the expression of *CBFs* and optimize plant fitness^[27,28]^. Similarly, hormones, including JA, BR, ethylene, and SL, interact with cold stress signaling in a CBF-dependent manner^[8]^. Despite these insights, the relationship between cold-responsive TFs and chromatin accessibility in woody perennials remains largely unexplored.

Chromatin accessibility reflects the degree of physical contact between nuclear macromolecules and DNA, serving as a primary determinant of genome organization and transcriptional capacity^[29,30]^. Transposase-accessible chromatin sequencing (ATAC-seq) has emerged as a robust tool for profiling accessible chromatin regions (ACRs) across various plant species and cell types with less labor input and high sensitivity compared to DNase-seq^[29,31]^. The integration of ATAC-seq and RNA-seq allows researchers to identify differentially expressed genes (DEGs) associated with promoter accessibility shifts, thereby uncovering core regulatory networks^[32−34]^. This approach has successfully identified TFs in wheat, rice, and the woody plant *Populus deltoides* × *P. euramericana* cv. 'Nanlin895'^[32−34]^. For example, comparative analysis in tomato identified WRKY34 as a pivotal cold-tolerance gene linked to differential chromatin accessibility^[35]^. Similarly, 13 TFs highly related to cold stress were identified in tea plant leaves integrating ATAC-seq and RNA-seq^[36]^. However, most studies have focused on leaf tissues. An integrated ATAC-seq and RNA-seq landscape of the shoot apex—the center of primary growth—remains poorly characterized.

Cold stress significantly impairs shoot apex development, including vertical growth, lateral organogenesis, and the vegetative-to-reproductive transition^[7,12]^. In temperate trees, apical buds enter dormancy to survive winter, thereby protecting the shoot apices from cold stress damage^[7]^. Conversely, in non-dormant tropical and subtropical plants, these apices remain active and vulnerable to cold stress^[7]^. For this reason, cold stress is one of the most important environmental stresses limiting the distribution and productivity of many tropical and subtropical woody plants^[7]^. *Eucalyptus* (*Myrtaceae*) encompasses three genera: *Eucalyptus*, *Angophora*, and *Corymbia*, with over 900 species/subspecies^[37]^. These perennial dicotyledonous trees or shrubs are native to Australia and Southeast Asia, with a natural distribution spanning from northern Australia to the Philippines^[38−40]^. *Eucalyptus* represents one of the most economically significant hardwood genera globally, accounting for approximately 23% of global plantation forests, and contributing over ⅓ of China's industrial timber output^[38−40]^. Known for their exceptional growth rates (up to 10 m per year) and short rotation periods (5 to 7 years), *Eucalyptus* is a primary source of timber and pulp and has been introduced to more than 95 countries and regions worldwide^[38−40]^. However, *Eucalyptus* productivity and cultivation range are severely constrained by low temperatures^[7]^. Cold stress during the coldest quarter acts as the dominant climatic constraint on the geographical distribution of *Eucalyptus dunnii*, with its contribution rate reaching 35.6%^[7]^. Cold stress significantly reduces the photosynthetic rate by decreasing the content of photosynthetic pigments (including chlorophyll) in *Eucalyptus*^[41,42]^. Additionally, it impairs cell membrane integrity, lowers water potential^[41]^, and consequently strongly inhibits leaf expansion, root elongation, and total biomass accumulation^[43,44]^. More importantly, cold stress causes the 'freeze-tip' phenomenon, where apical buds and young shoots sustain lethal injury, leading to significant yield losses^[45−47]^. While the transcriptomic, proteomic, and metabolomic responses of *Eucalyptus* leaves have been documented^[48,49]^, the molecular mechanisms governing shoot apex cold resilience—and the role of chromatin accessibility therein—remain elusive.

In this study, we performed a high-resolution time-series analysis of cold stress responses in the shoot apices of *Eucalyptus grandis* (*E. grandis*) by integrated ATAC-seq and RNA-seq. We clarified the temporal hierarchy of chromatin remodeling and transcriptional reprogramming, and identified key TFs that potentially mediate the 'development-to-stress' switch. This research provides a fundamental theoretical framework and genetic resources for the improvement of cold tolerance in *Eucalyptus* and other subtropical woody species.

## Materials and methods

### Plant materials and growth conditions

*E. grandis* clone Eg5 tissue-cultured seedlings were provided by Prof. Wei Wu from the Seedling Center of Fujian Yong'an Forestry Group Co., Ltd. Briefly, axillary buds were subcultured on Murashige and Skoog (MS) medium (supplemented with 0.4 mg/L 6-BA, 0.2 mg/L NAA, 30 g/L sucrose, 0.05 g/L cysteine, and 8 g/L agar; pH 5.8) for 25 d. Rooting of these axillary buds was conducted on 1/2 MS medium (supplemented with 0.5 mg/L IBA, 15 g/L sucrose, 7 g/L carrageenan, and 0.05 g/L cysteine; pH 5.8) for 10 d. The rooted seedlings were transplanted into soil and cultivated in a growth chamber for four weeks under the following conditions: light intensity of 50 μmol/m^2^/s, relative humidity of 60%, a 16 h light/8 h dark photoperiod, and a temperature of 25 °C. Seedlings of uniform vigor were selected for a time-series cold stress experiment, where they were exposed to 4 °C for 0, 0.5, 2, 6, 12, and 24 h. For transcriptome analysis, 15 shoot apices were pooled per biological replicate; RNA-seq was performed in triplicate. For ATAC-seq, two fresh shoot apices were pooled per biological replicate, with three biological replicates maintained for each time point.

### Phenotypic analysis

Seedlings of the same age as those used in the molecular analysis were subjected to 4 and 25 °C for 0.5 and 24 h, respectively. Following the treatment, seedlings were transferred to a 25 °C growth chamber for a 3-d recovery period under identical baseline conditions to those used in the transcriptome analysis. Morphological observations were conducted before treatment, immediately after treatment, and after the recovery period. Images were acquired using a Leica S9i stereomicroscope at 2× magnification (parameters: gain = 4×, saturation = 60, and gamma = 0.60). Shoot apex length was measured using ImageJ software.

### ATAC-seq library construction and sequencing

Fresh shoot apices were chopped into a homogenate on a pre-chilled Petri dish. The homogenate was resuspended in lysis buffer (15 mM Tris-HCl, pH 7.5; 4 mM NaCl; 0.2 mM spermidine; 0.5 mM DTT; 0.2% Triton X-100), and incubated at 4 °C for 10 min. The lysate was filtered through two layers of Miracloth (Merck, Cat. No. 475855-1R). The filtrate was layered onto a sucrose density buffer (20 mM Tris-HCl, pH 8.0; 2 mM MgCl_2_; 2 mM EDTA; 7.5 mM DTT; 0.2% Triton X-100; 1.2 M sucrose) and centrifuged at 2,200 × *g* (4 °C) for 20 min. The nuclear pellet was washed with pre-chilled wash buffer (10 mM Tris-HCl, pH 8.0; 5 mM MgCl_2_), and pelleted by centrifugation at 1,200 × *g* for 4 min. Purified nuclei were transposed using Tn5 transposase following the manufacturer's instructions (Vazyme, Cat. No. 12207ES96). The tagged product was immediately purified using two volumes of DNA Clean Beads (Vazyme, Cat. No. N41102). Purified chromatin fragments were processed for DNA library preparation with the TruePrep DNA Library Prep Kit V2 (Vazyme, Cat. No. TD50102) and TruePrep Index Kit V2 (Vazyme, Cat. No. TD202). Subsequently, size selection was performed using 0.55×, and 1× volumes of DNA Clean Beads. Final ATAC-seq libraries were sequenced on the BGI DNBSEQ-T7 platform (150 bp paired-end reads) by Berry Genomics Co., Ltd (Beijing, China).

### RNA-seq library construction and sequencing

Total RNA was extracted from *E. grandis* shoot apices using the Polysaccharide Polyphenol Total RNA Extraction Kit (TIANGEN, Cat. No. A1104A). Oligo d(T)_25_ magnetic beads (NEB, Cat. No. S1419S) were washed with binding buffer, resuspended, and incubated with 500 ng of RNA for 5 min at room temperature. The beads were then collected, washed twice with washing buffer, and poly(A) + RNA was eluted by heating with DEPC-treated water (Sangon Biotech, Cat. No. B501005) at 80 °C for 3 min. The eluted poly(A) + RNA was fragmented at 94 °C for 3 min using 5 × First Strand Buffer (TaKaRa, Cat. No. 639536). Complementary DNA (cDNA) was synthesized with SMARTScribe Reverse Transcriptase (TaKaRa, Cat. No. 639536) and an RNase Inhibitor (Vazyme, Cat. No. R301-03) following the manufacturer's protocol, then purified with an equal volume of DNA Clean Beads (Vazyme, Cat. No. N41102). Purified cDNA was used for library preparation with the KAPA HiFi Kit (KAPA Biosystems, Cat. No. KK2101). The size selection was executed using 0.6 × and 0.2 × volumes of DNA Clean Beads. Final libraries were sequenced on the Illumina NovaSeq platform (150 bp paired-end reads) by Berry Genomics Co., Ltd.

### Bioinformatics data processing

Quality filtering and adapter trimming of raw reads were performed using fastp (v0.22.0, -q 20 -l 18). For ATAC-seq, clean reads were aligned to the *E. grandis* genome (NCBI Genome Assembly ASM1654582v1) using Bowtie2 (v2.3.5.1)^[38]^. PCR duplicates were identified and removed using Picard MarkDuplicates (v1.141) with the ASSUME_SORTED = true, and REMOVE_DUPLICATES = true options. To account for the characteristics of Tn5 transposase, mapped reads were shifted using the alignmentSieve function in deepTools (v3.5.4)^[50]^. Chromatin accessibility regions (ACRs) were identified using MACS3 (-g 626634579, --keep-dup all, and -q 0.05)^[51]^. Peak abundance was quantified using DiffBind (v3.16.0) with minOverlap = 1. Time-series differential ACRs (dACRs) were identified using the Likelihood Ratio Test (LRT) implemented in the DESeq2 package (v1.46.0) with a threshold of *p* < 0.05. For each dACR, the time point with the highest peak count was defined as the high dACR, whereas the time point with the lowest peak count was defined as the low dACR. Pairwise differential ACRs relative to the 0 h control were identified using DiffBind with a threshold of *p* < 0.01.

For RNA-seq, clean reads were mapped to the *E. grandis* genome using STAR (v2.5.2b) and quantified with featureCounts (v2.0.1)^[52]^. Differentially expressed genes (DEGs) between each time point and the 0 h control were identified via DESeq2 with thresholds of *p* < 0.01, and |log_2_FoldChange| ≥ 1. Gene ontology annotation for *E. grandis* genes was conducted using eggNOG-mapper (v5.0)^[53]^, and GO enrichment analysis was performed using clusterProfiler (v4.14.6) in R^[54]^.

### Motif analyses and target site scanning

High dACR coordinates were analyzed using findMotifsGenome.pl (HOMER suite) for *de novo* motif discovery^[55]^. The parameters were set to -size 50, -mset plants, and -bg using the low dACR coordinate list as background. Potential false-positive motifs were filtered and excluded from downstream analyses. Odds ratios were calculated as the ratio of the percentage of motif-contained targets to that in the background, reflecting the strength of motif enrichment. The match score between *de novo* identified motifs and known plant motifs represented their degree of similarity, with 0.6 considered an acceptable threshold. For motif variability calculation, identified motifs were imported into R and computed using ChromVar with set.seed (19960203). The accessibility correlation between pairs of TF motifs was calculated using the hierarchical clustering (hclust) method. Furthermore, synergy levels between motif pairs were assessed to indicate potential cooperative or competitive binding events in accessible chromatin regions. Target genes containing TF motifs were identified using the scan_sequences function in the universalmotif R package with logodds ≥ 0.6, RC = TRUE, and no.overlaps = TRUE.

### Luciferase reporter assay

For motif activity assays, eight tandem repeats of each motif were synthesized and cloned into the pGreenII 0800-LUC vector. Plasmids were extracted using an Endofree Maxi Plasmid Kit (TIANGEN, Cat. No. DP117). The CaMV 35S promoter and mini-35S promoter were used as controls. The resulting constructs were transformed into *Arabidopsis* protoplasts isolated from 3−4 week old seedlings. After overnight expression, protoplasts were subjected to 4 °C for 0.5 h, followed by a 0.5 h recovery at room temperature to restore translational activity before the luciferase assay. Luciferase activity was quantified using a dual-luciferase reporter assay kit (Beyotime, Cat. No. RG089S). Firefly luciferase (FLuc) and Renilla luciferase (RLuc) signals were recorded using a BioTek Cytation 5. FLuc signals were normalized to RLuc signals and further normalized to the non-stressed control. Three replicates were performed for each treatment.

For transcription factor–promoter interaction assays, a 2 kb promoter fragment of *CBF4* (*LOC104449076*) was cloned into the pGreenII 0800-LUC vector. Two candidate *AGL42* transcription factor coding sequences were cloned into the HBT-Flag vector. The resulting constructs were co-transfected into *Arabidopsis* protoplasts and expressed overnight, followed by cold stress treatment as described above.

All primers and synthetic motifs used in this study are listed in Supplementary Table S1.

### Homologous gene search

An *Arabidopsis* protein BLASTp database was generated via makeblastdb. Protein sequences corresponding to cold-responsive dACRs in *E. grandis* were queried against this database (BLASTp; -evalue = 1e–5, and -max_target_seqs = 1). Homologous genes were identified using an E-value threshold of < 1e–10, and sequence identity of > 50%.

## Results

### Dynamic chromatin accessibility under cold stress in *E. grandis* shoot apices

**Figure 1:**
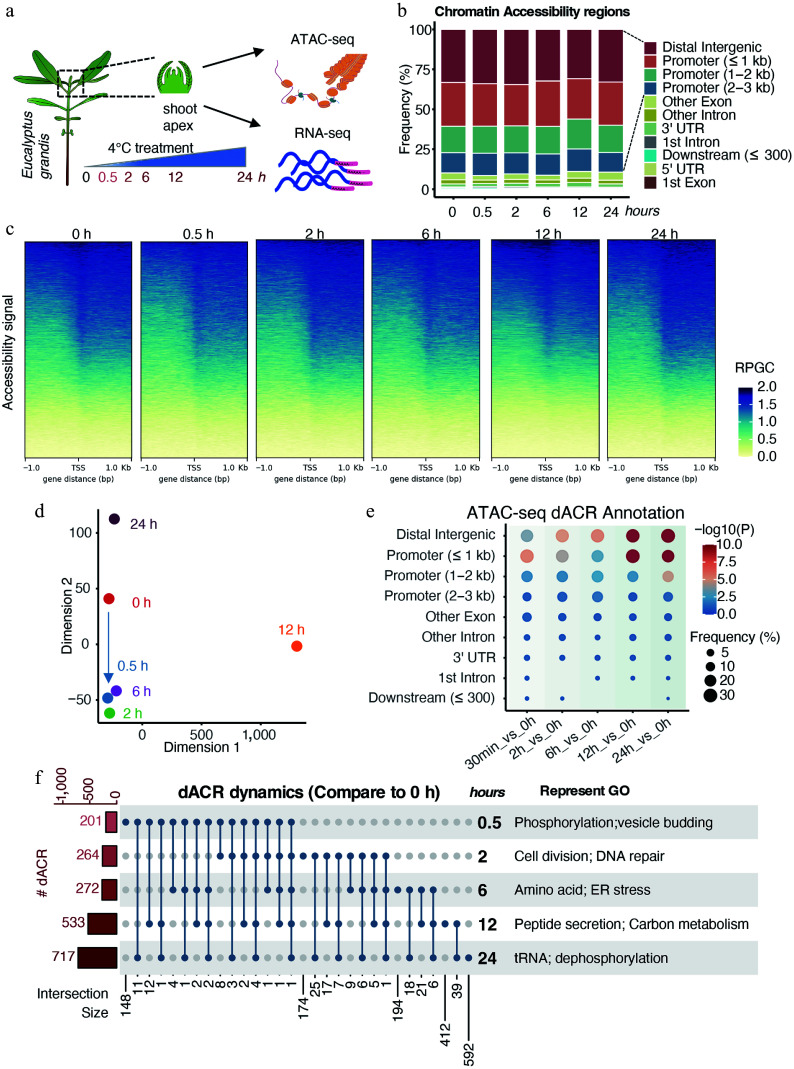
Dynamic chromatin accessibility of *E. grandis* under cold stress. (a) Experimental scheme of sampling in this study. (b) Annotation of ATAC-seq peaks. (c) Chromatin accessibility signals around transcription start sites (TSS). Signals were calculated using reads per genomic content (RPGC) normalization. (d) Multidimensional scaling (MDS) analysis of samples. (e) Distribution of differentially accessible regions (dACRs) compared with the 0 h control. (f) Upset plot and GO enrichment analysis of genes associated with dACRs.

Cold stress imposes a pronounced adverse effect on shoot growth and development in *Eucalyptus* species^[45]^. Genome architecture and chromatin accessibility represent key determinants of cellular functional states^[56]^. To dissect the dynamics of chromatin accessibility triggered by cold stress, we performed an assay for transposase-accessible chromatin sequencing (ATAC-seq) on shoot apices of *E. grandis* across a 0–24 h cold stress time course (Fig. 1a). These accessible chromatin regions (ACRs) were widely distributed across the genome, with the highest enrichment at promoter regions, and signal clustering concentrated around transcription start sites (Fig. 1b, c). These patterns affirmed the reliability of our experimental data.

To better characterize differences in chromatin accessibility across samples, Multidimensional Scaling (MDS) analysis revealed rapid chromatin accessibility remodeling within 0.5 h of cold stress, with similar profiles maintained at 2 and 6 h (Fig. 1d). In addition, chromatin states at 12 and 24 h diverged markedly from the 0 h control and early stress time points, indicating extensive cold-induced chromatin reprogramming.

To elucidate the chromatin accessibility dynamics induced by cold stress, we conducted differential accessibility chromatin region (dACR) analyses by comparing each time point with the 0 h control. Results indicated that dACRs were primarily located in distal intergenic and promoter (≤ 1 kb) regions (Fig. 1e). Notably, the enrichment (Fisher's exact test) and number of dACR-associated genes showed a steady upward trend, rising from 201 at 0.5 h, to 717 at 24 h (Fig. 1e, f). These dACRs also exhibited strong temporal specificity. Gene Ontology (GO) enrichment analyses of genes harboring dACRs revealed a distinct temporal trajectory that mirrored the duration of cold treatment (Fig. 1f; Supplementary Fig. S1a). Briefly, enrichment identified stage-specific processes: protein phosphorylation and vesicle budding at 0.5 h; cell division and DNA repair at 2 h; amino acid metabolism and ER stress at 6 h; and carbon metabolism and protein dephosphorylation at 12–24 h (Supplementary Table S2). Together, these results demonstrate a rapid initial chromatin response followed by progressive, time-dependent remodeling under sustained cold stress.

### Rapid transcriptomic reprogramming of shoot apices under cold stress

**Figure 2:**
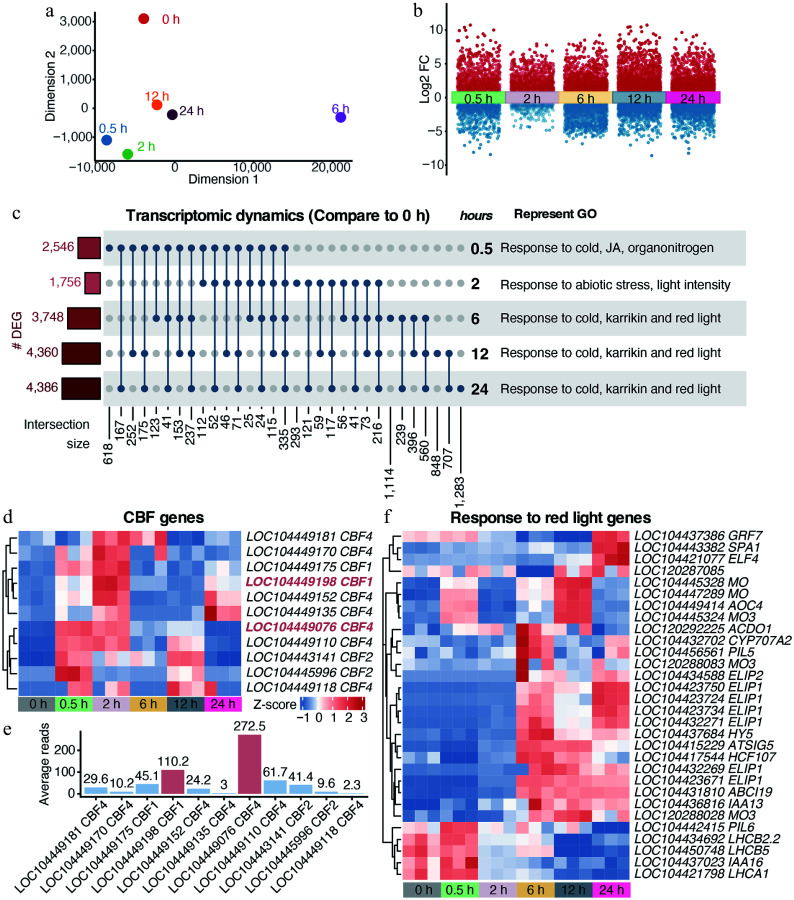
Rapid transcriptomic reprogramming of *E. grandis* under cold stress. (a) MDS trajectory of RNA-seq data. (b) Transcriptomic dynamics at different time points under cold stress compared with the 0 h control. (c) Covariation and GO enrichment analysis of differentially expressed genes (DEGs) compared with the 0 h control. (d) and (e) Dynamic expression of putative *CBFs* in *E. grandis.* (f) Cold stress reprograms the expression of genes responsive to red light.

Given its role in modulating transcription factor access, chromatin accessibility is crucial for transcriptional reprogramming. Accordingly, parallel time-series RNA-seq on the shoot apex samples was performed. MDS analysis showed that transcriptomic changes were evident as early as 0.5 h (Fig. 2a). Transcriptomic divergence became most pronounced at 6 h, which was clearly separated from all other time points. Although partial convergence was observed at 12 and 24 h, the transcriptomic states remained distinct from the 0 h control, indicating sustained reprogramming. This reprogramming was substantial, with numerous genes showing pronounced expression shifts (Fig. 2b). Differentially expressed genes (DEGs) displayed strong temporal specificity, consistent with chromatin dynamics, suggesting stress intensity-dependent regulation of cold stress responses (Fig. 2c). GO enrichment revealed a shift from cold- and JA-responsive pathways to red light–associated processes (Fig. 2c; Supplementary Fig. S1b; Supplementary Table S2).

CBFs serve as core transcription factors that orchestrate the transcription of *Cold-Regulated* (*COR*) genes in the plant cold stress signaling pathway^[8,21,57−60]^. Based on *Arabidopsis* CBFs, we identified *CBF* homologs in *E. grandis*. Expression profiling showed that most *E. grandis* *CBFs* were rapidly induced within 0.5–2 h (Fig. 2d). Notably, *LOC104449076* (putative *CBF4*) and *LOC104449198* (putative *CBF1*) exhibited the highest expression levels (Fig. 2e), suggesting they act as primary regulators. Conversely, most red-light-responsive genes were activated at later stages (6–24 h, Fig. 2f), while photosystem-related genes, such as the *LHCB* gene, were notably repressed, suggesting suppressed photosynthetic capacity under cold stress.

In summary, dynamic changes in the transcriptome, as well as chromatin accessibility, revealed a coordinated and rapid response of the *E. grandis* shoot apices to cold stress, characterized by early CBF activation and subsequent secondary responses involving reduced photosynthetic activity and induction of red light-responsive pathways.

### Time-lapse chromatin dynamics induced by cold stress

**Figure 3:**
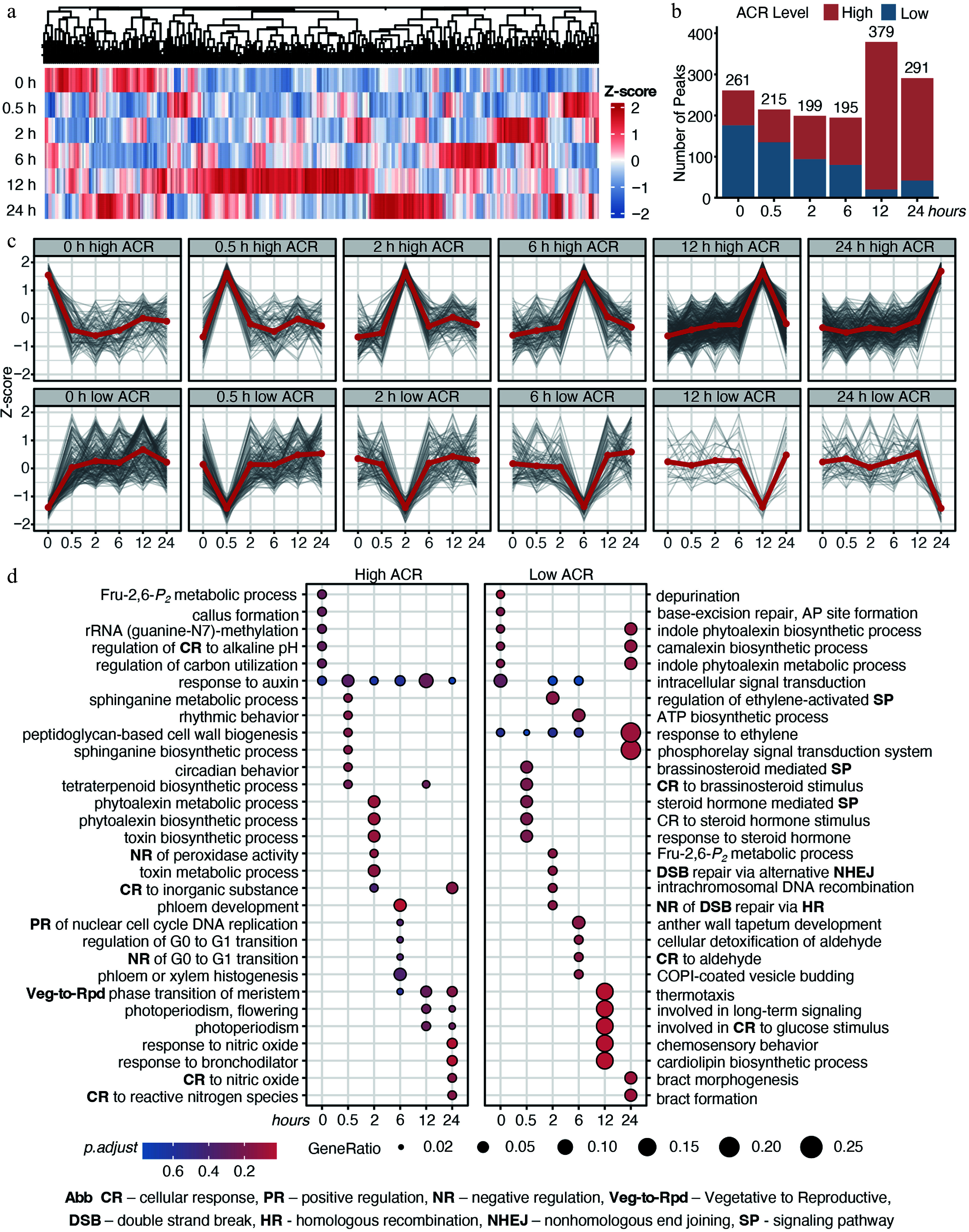
Time-lapse chromatin accessibility dynamics of *E. grandis* during cold stress. (a) Time-series significant chromatin accessibility dynamics in the shoot apex. (b) Counts of high- and low-accessibility peaks. (c) Trends of high- and low-accessibility peaks across cold-stress periods. (d) GO enrichment analysis of genes with significant chromatin accessibility peaks. Abbreviations are indicated at the bottom of the figure.

To profile temporal accessibility, we identified 1,345 differentially accessible chromatin regions (dACRs, *p* < 0.05) via likelihood ratio tests (LRT) (Fig. 3a; Supplementary Table S3). Each dACR was classified as a 'high ACR' at its peak accessibility and a 'low ACR' at its minimum. We observed a progressive increase in high ACRs with prolonged stress, accompanied by a decrease in low ACRs (Fig. 3b), indicating significant chromatin opening. The classifications and accessibility trends were confirmed again by the temporal dynamics of high and low ACR clusters independently (Fig. 3c).

To decipher the biological function of these stage-specific ACR dynamics, we performed Gene Ontology (GO) enrichment analysis on genes with high and low ACRs (Fig. 3d). High ACRs displayed clear temporal shifts in functional enrichment during cold stress (Fig. 3d left panel; Supplementary Table S4). As early as 0.5 h, chromatin opening occurred at auxin-responsive and circadian-related genes. By 2 h, high ACRs were enriched for phytoalexin metabolism and peroxidase activity regulation, followed by cell cycle regulation and vegetative-to-reproductive phase transition of the meristem at 6 h. At 12–24 h, high ACRs were associated with nitric oxide and light signaling. Interestingly, auxin-responsive genes exhibited regulated chromatin accessibility across multiple time points, highlighting their sustained involvement throughout cold stress. In contrast, low ACRs were enriched for ethylene and brassinosteroid (BR) signaling at early stages and long-distance signaling at late stages (Fig. 3d right panel; Supplementary Table S4).

Collectively, these results demonstrated that cold stress induced stage-specific, functionally divergent changes in chromatin accessibility in *E. grandis* shoot apices. Particularly, during the early response phase, cold stress rapidly increased chromatin accessibility in auxin-responsive genes, while concurrently reducing accessibility in genes involved in ethylene and BR signaling, indicating a coordinated role of hormone signaling in cold stress response in the shoot apices.

### Chromatin accessibility was associated with transcriptional dynamics under cold stress

**Figure 4:**
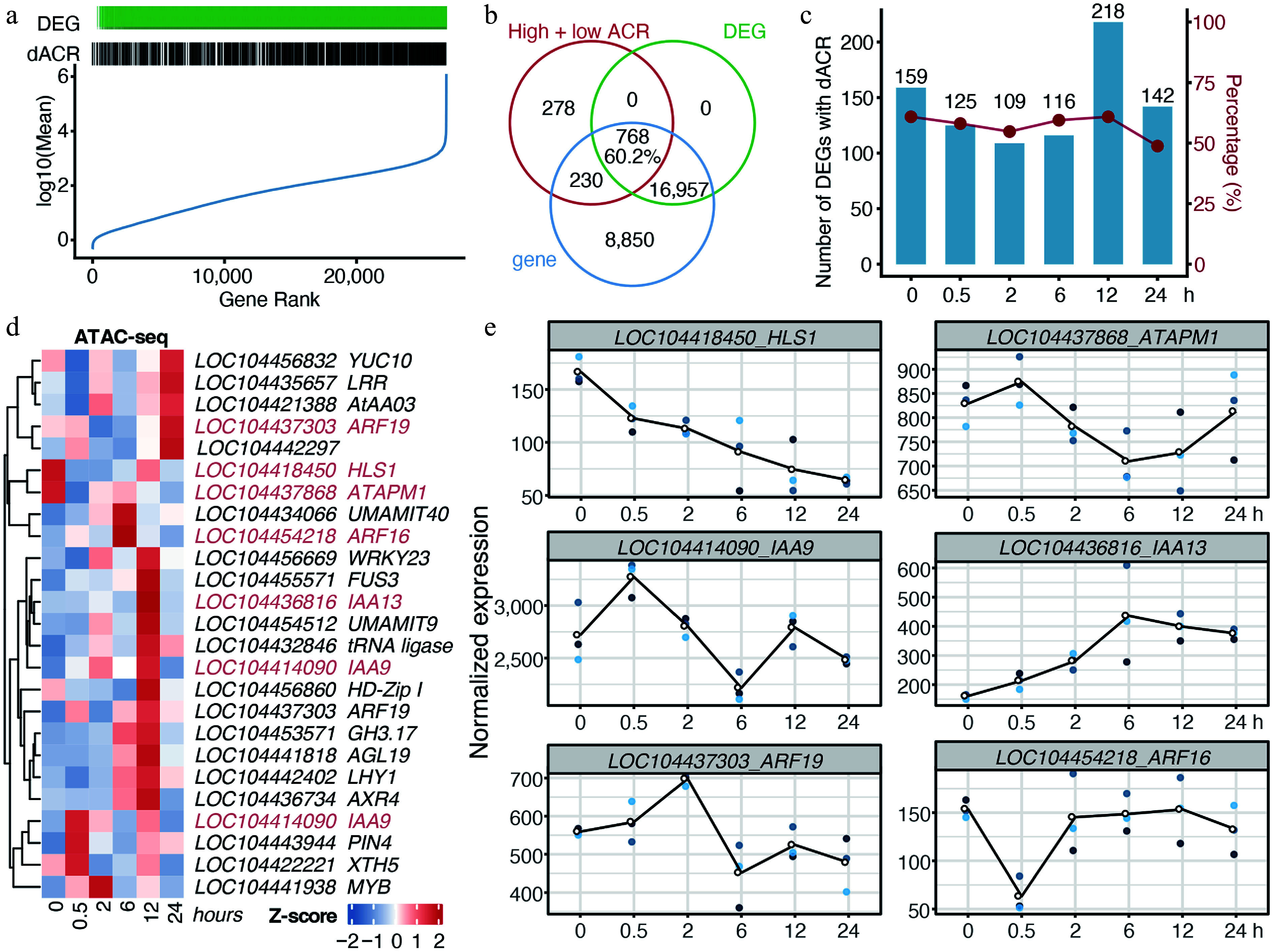
Chromatin accessibility dynamics under cold stress were associated with transcription. (a) Distribution of genes with differentially chromatin accessible regions (dACRs) and/or differential expression (DEGs). Genes were ranked from low to high average expression. (b) Venn diagram of genes with dACRs, DEGs, and total genes identified in RNA-seq. (c) Counts and proportions of DEGs harboring dACRs. (d) Chromatin accessibility of genes involved in the 'response to auxin' GO term identified in Fig. 3d. Homologs of *Arabidopsis* genes are indicated after loci IDs. (e) Gene expression dynamics of auxin-responsive genes identified from RNA-seq data. Each time point includes three biological replicates, indicated by dots.

Time-course DEGs were also identified by the LRT method (Supplementary Fig. S2). The dACRs and DEGs were not restricted to genes with extreme expression levels (Fig. 4a), confirming that cold stress elicited genuine chromatin and transcriptional reprogramming. Venn analysis showed that 60.2% of genes harboring dACRs were classified as DEGs (Fig. 4b). This strong association was maintained across time points (Fig. 4c), indicating a tight coupling between chromatin accessibility and gene expression.

Given our previous observation that the auxin response module was persistently activated under cold stress (Fig. 3d), we profiled the temporal chromatin accessibility and gene expression dynamics of auxin-related genes identified via GO analysis to evaluate the link. Cold stress rapidly suppressed the chromatin accessibility of *E. grandis* homologs of *Arabidopsis* *HLS1* (*LOC104418450*) and *ATAPM1* (*LOC104437868*) (Fig. 4d), with a corresponding reduction in their transcript levels (Fig. 4e). For *IAA* family homologs (e.g., *IAA9*, *IAA13*), transcriptional changes were synchronous with chromatin dynamics, while *ARF* family homologs showed consistent overall expression trends despite less tightly matched patterns, supporting a general coupling between chromatin remodeling and transcriptional regulation under cold stress.

To further clarify how chromatin remodeling events (e.g., pre-stress and immediate post-stress) correlate with temporal transcriptional regulation, we analyzed the expression landscape of all cold-induced high accessible chromatin region (ACR) genes. Genes with pre-existing high ACRs at 0 h represent constitutively open chromatin in the shoot apices (Fig. 3c), and they exhibited two distinct expression patterns: native high-expression genes were gradually downregulated after cold stress (Supplementary Fig. S3, cluster 1 of 0 h), while a large subset displayed time-dependent stress-induced expression (rapid: 0.5 h; intermediate: 2–6 h; late: 12–24 h) (Supplementary Fig. S3, cluster 2–5 of 0 h). Temporal asynchrony between chromatin accessibility and gene expression was evident in 0.5 h high ACR genes, which showed native high-expression genes (e.g., *PIN1*) transiently downregulated at 0.5 h, whereas auxin signaling (e.g., *IAA9*) and nutrient transport (e.g., *YSL2*, *NPF2.9*) genes showed delayed expression changes. Similar asynchronous patterns were observed for high ACRs identified at 2–24 h (Supplementary Fig. S3).

Collectively, these results delineated a temporal blueprint whereby chromatin accessibility responses with different kinetics shapes time-dependent transcriptional regulation under cold stress.

### Chromatin-based hierarchical gene regulation networks under cold stress

**Figure 5:**
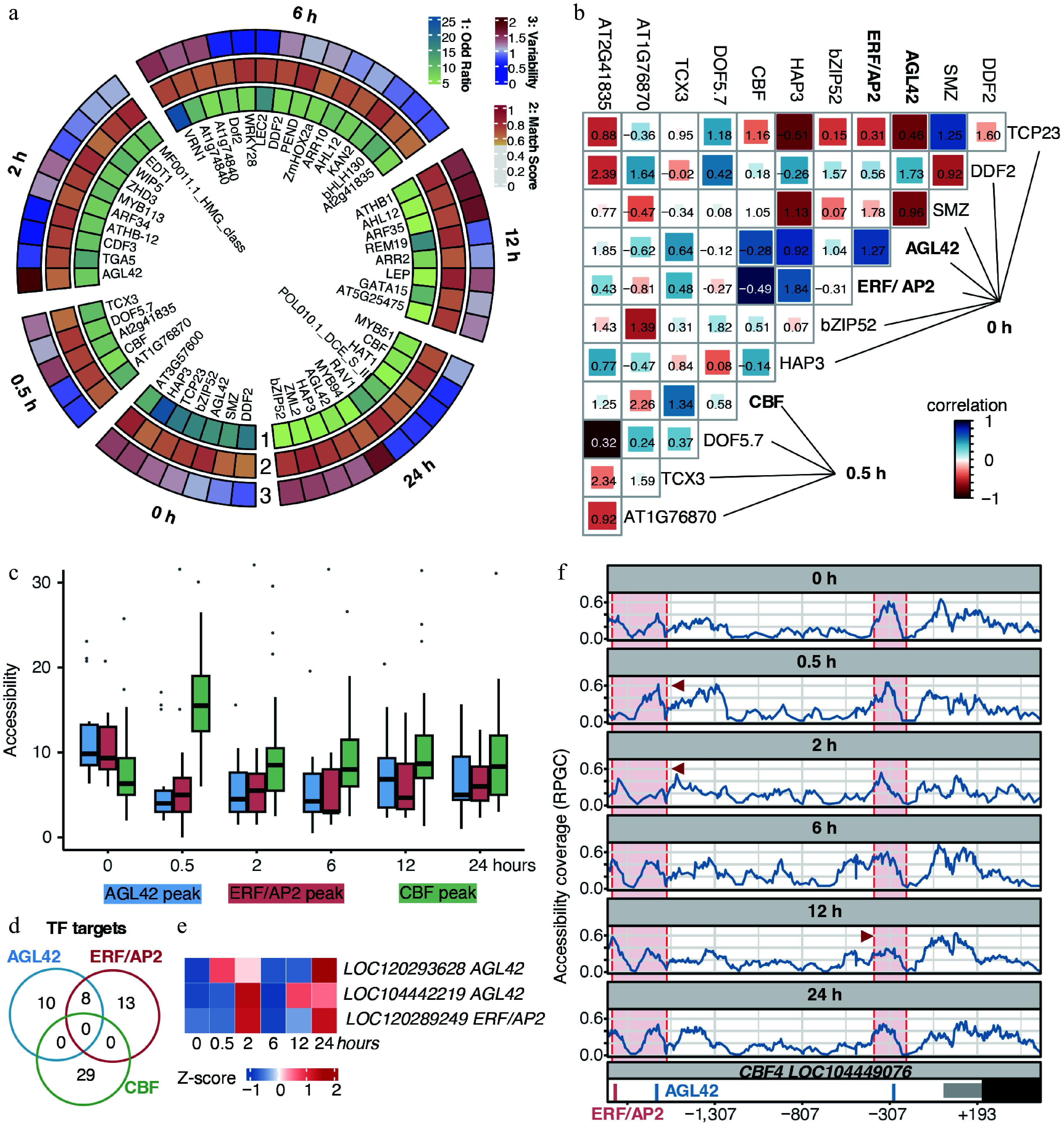
Chromatin-mediated hierarchical gene regulatory networks of *E. grandis* during cold stress. (a) Motifs were identified using the Homer suite. Only motifs with *p-*values higher than the threshold for potential false positives were retained. The Odd ratio was calculated as the percentage of motif-containing targets, divided by the corresponding percentage in the background. Match scores were obtained by comparison with the best-matched transcription factor motifs. Motif variability was calculated using chromVar to reflect motif specificity in the corresponding samples. (b) Chromatin co-accessibility and co-localization analyses between putative TF pairs. Color indicates the correlation coefficient, representing chromatin co-accessibility of TF target regions. Numbers in the figure indicate co-binding scores of putative TFs. (c) Chromatin accessibility level of regions targeted by AGL42-, ERF/AP2-, and CBF-like TFs. (d) Venn diagram of potential target genes of AGL42-, ERF/AP2-, and CBF-like TFs. (e) Time-course gene expression of candidate *AGL42* and *ERF/AP2* genes. (f) Chromatin accessibility and TF binding sites in the putative *CBF4* promoter. Regions enclosed by red shading indicate the vicinity of AGL42 and ERF/AP2 target sites, and positions indicated by red arrows represent regions with significant coverage changes.

To dissect TF dynamics under cold stress in *E. grandis* shoot apices, we performed a HOMER motif enrichment on high ACRs using low ACRs as background (Supplementary Fig. S4; Supplementary Table S5). At 0 h, seven motifs were identified (Fig. 5a), corresponding to TFs in shoot apex development (e.g., DDF2, SMZ, AGL42, bZIP52, TCP23, HAP3, and AT3G57600 [ERF/AP2])^[61−65]^, indicating that our data was reliable and accurate. At 0.5 h, motifs for stress-responsive TFs, including CBF, TCX3, and DOF5.7^[8,66]^ were enriched, confirming rapid chromatin remodeling. At later time points (2–24 h), motif enrichment analysis of high ACRs revealed the progressive engagement of potential transcription factors associated with stress responses (e.g., WIP5), auxin signaling (ARF), and chromatin regulation (AHL) (Fig. 5a). Notably, the transcription factor motif of LEC2, a master regulator with the capacity to reprogram cell fate^[67]^, was significantly enriched at 6 h. Given that the transcriptome diverged most at 6h (Fig. 2a), this suggests a potential transition from acute stress signaling to developmental reprogramming. This sequential activation of TFs reflected a progressive transition of the shoot apices from a growth/development-oriented state to rapid cold stress response, accompanied by chromatin remodeling and potential cell fate transition.

Next, we used chromVAR-derived motif variability to calculate correlations in chromatin accessibility between motifs and to analyze their co-localization patterns. We observed that certain TF pairs exhibited strong correlations in chromatin accessibility at their binding sites (Supplementary Fig. S5). For example, at 0 h, AGL42 and AT3G57600 showed high positive correlations (blue color) with most TFs responding along the stress time course. At 2 h, ARF34 and AGL42 displayed strong correlations with TFs activated at 12−24 h of stress, while at 6 h, AT1G74840 and WRKY family TFs were also highly correlated with TFs responding at 12−24 h. In contrast, the co-accessibility of binding sites for some TFs was predominantly negatively correlated (red color). For instance, TCP23 at 0 h exhibited strong negative correlations with multiple stress-responsive TFs, and REM19 at 12 h was negatively correlated with TFs activated at later stages of the stress response. Motif co-localization analysis further revealed a complex regulatory network of interactions between these putative TFs and their targets (values on the figure). Thus, chromatin co-accessibility and motif co-localization features reflected the dynamic and hierarchical interplay of chromatin organization and transcriptional regulation in the *E. grandis* shoot apices under cold stress.

We next focused on the 0−0.5 h window to illustrate the TFs' hierarchical network during the rapid cold response. We found that, in the shoot apices at 0 h, locally expressed AGL42- and ERF/AP2-like TFs exhibited pronounced chromatin co-accessibility with CBF target regions activated at 0.5 h (Fig. 5b). Results suggested two possible mechanisms underlying rapid cold response: 1) these TFs might co-bind to the same targets, 2) *CBF* in shoot apices was regulated by AGL42- and ERF/AP2-like TFs. Low co-localization scores suggested that AGL42- and ERF/AP2-like TFs might not directly co-bind DNA with *CBFs* (Fig. 5b). Then, we checked the chromatin accessibility levels of the target regions of the 3 TFs. We found that, at 0 h, the chromatin accessibility of AGL42- and ERF/AP2-like TF target regions was substantially higher than that of CBF target regions, whereas this trend was dramatically reversed at 0.5 h (Fig. 5c). Moreover, genes located downstream of the target regions of these TFs did not overlap (Fig. 5d). Thus, the temporal reversal in chromatin accessibility suggested that AGL42 and ERF/AP2-like TFs might function upstream of *CBFs* during the early cold response. BLAST suggested that the *AGL42* homologs in *E. grandis* were *LOC120293628* and *LOC104442219*, and that the *ERF/AP2* homolog is *LOC120289249* (Fig. 5e; Supplementary Fig. S6; Supplementary Table S6). Importantly, the *CBF4* promoter contains two AGL42 binding motifs and one ERF/AP2 binding motif (Fig. 5f). Notably, the *CBF4* promoter exhibited an early chromatin-primed, or permissive chromatin state, and underwent a rapid increase in chromatin accessibility upon cold stress, with the dynamic accessible regions coinciding with the AGL42 and ERF/AP2 binding motifs. Collectively, we revealed a gene regulatory network underlying the rapid CBF-mediated response to cold stress in the shoot apices.

### Cold stress rapidly inhibited shoot apex growth

**Figure 6:**
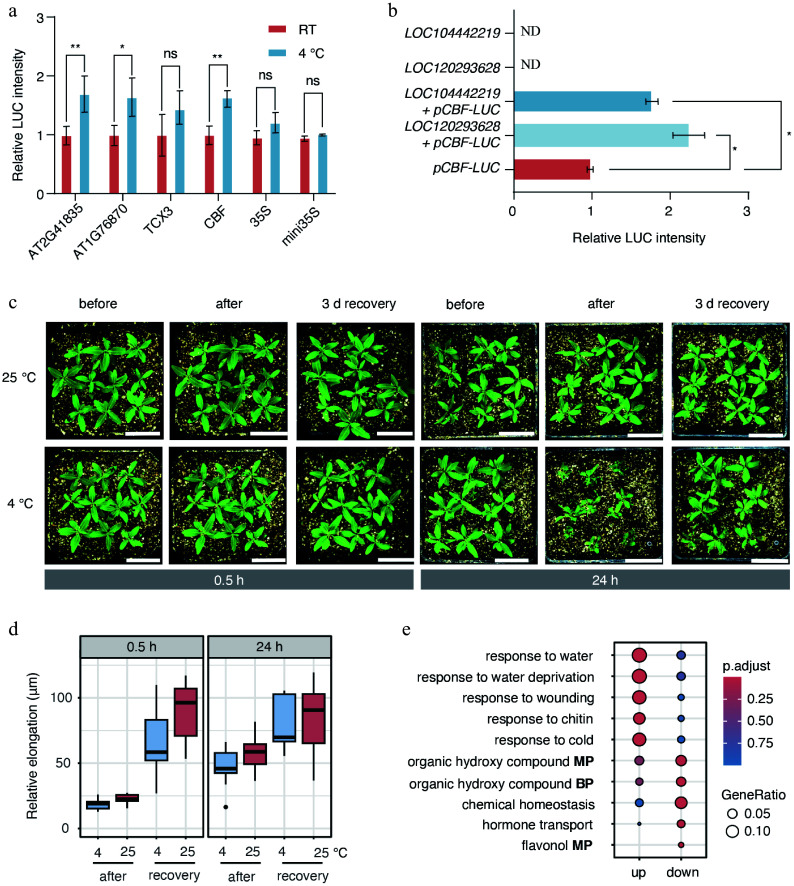
Cold stress inhibited shoot apex growth. (a) Dual-luciferase assays were used to measure the activity of motifs identified from ATAC-seq data under 0.5 h cold-stress conditions. RT indicates room-temperature control. Statistical significance was evaluated using analysis of variance (ANOVA). *p* < 0.05 and *p* < 0.01 are indicated by * and **, respectively. (b) Interaction between the putative transcription factor AGL42 and the promoter of the putative *CBF4* gene (*LOC104449076*). 'ND' indicates not detected. (c) Growth of *E. grandis* seedlings before cold stress, after cold stress, and after 3 d of recovery. Bar = 3 cm. (d) Relative elongation length after treatment and/or recovery compared with the pre-treatment status. (e) GO enrichment analysis of differentially expressed genes between the 0.5 h stress group, and the 0 h control group. BP indicates 'biological process'; MP indicates 'metabolic process'.

To assess the impact of transient cold stress on *E. grandis*, we validated four rapid cold-responsive motifs (0.5 h) identified from ATAC-seq, which exhibited clear sequence logos (Supplementary Fig. S4), using dual-luciferase reporter assays. All four motifs increased LUC activity, with *CBF*, *AT1G76870,* and *AT2G41835* motifs showed significant induction (Fig. 6a). We next examined whether the putative AGL42 of *E. grandis* could activate *CBF* expression under cold stress. Furthermore, co-expression of two putative *AGL42* (*LOC120293628* and *LOC104442219*) significantly enhanced *CBF4* (*LOC104449076*) promoter-driven LUC activity (Fig. 6b), confirming that AGL42 regulates *CBF4* during cold stress.

We further examined changes in shoot apex size under cold stress and after a 3 d recovery period. It was not surprising that no significant change in shoot apex length was observed right after 0.5 h cold stress (Fig. 6c, d). However, after 3 d of recovery, shoot apex elongation was markedly inhibited by the prior cold stress (Fig. 6c, d). The 24 h cold treatment resulted in a clear suppression of shoot apex elongation immediately after stress exposure. These findings indicated that even short-term cold stress exerted a substantial disruptive effect on shoot apex growth and development in *E. grandis*.

Furthermore, GO analysis of DEGs between the 0.5 h cold treatment and the 0 h showed that upregulated genes were predominantly enriched in stress-response–related processes, whereas downregulated genes were mainly associated with secondary metabolic processes and hormone transport (Fig. 6e). This suggests that the shoot apices rapidly downregulated secondary metabolic processes under cold stress to resist or adapt to the stress. These results further supported the existence of a rapid cold-responsive regulatory program in the *E. grandis* shoot apices.

Overall, our study revealed that at the levels of chromatin remodeling and transcriptional regulation, a rapid cold-stress–responsive signaling network and its potential underlying mechanisms in the *E. grandis* shoot apex.

## Discussion

As a globally important commercial forest species, *Eucalyptus* species are strongly constrained by cold stress, which limits both their productivity and geographical distribution^[7,39,40]^. Unlike seasonally dormant temperate trees, *Eucalyptus* species are directly exposed to sudden cold waves during low-temperature seasons, often resulting in abrupt growth arrest and dehydration^[68]^. The transcriptional regulation networks within shoot apices are known to play a critical role in cold stress response and subsequent annual growth arrest^[69]^. Although previous studies have reported that chromatin states and gene expression are regulated by cold stress in woody species^[70]^, to our knowledge, this study provides the first evidence that the shoot apices undergo highly coordinated chromatin and transcriptional reprogramming upon acute cold exposure (Figs 1−3). By integrating time-resolved ATAC-seq and RNA-seq, we elucidated the synchronized dynamics between chromatin accessibility and transcription (Figs 4, 5), revealing the regulatory logic governing the cold stress response in *E. grandis*.

**Figure 7:**
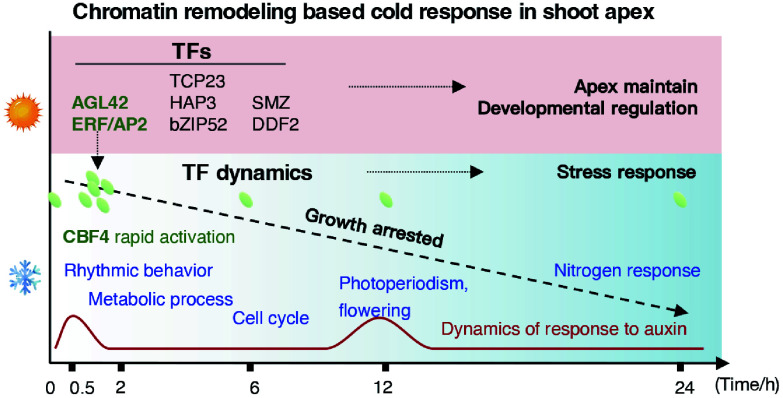
Temporal hierarchy of chromatin accessibility and transcriptional regulation in *E. grandis* in response to cold stress.

Notably, the rapid chromatin remodeling observed within 0.5 h of cold exposure in *E. grandis* shoot apices constitutes a classic hypersensitive stress response, which appears adaptive for non-dormant subtropical woody perennials (Fig. 7). While temperate trees have evolved to pre-establish dormant states to withstand predictable seasonal cold^[4,7]^, *E. grandis* lacks an inherent winter dormancy mechanism. Consequently, it relies on near-instantaneous, genome-wide chromatin transitions to initiate signaling cascades upon sudden cold-wave exposure. This hypersensitive chromatin response was characterized by the immediate opening of ACRs associated with hormone and circadian signaling genes (Fig. 3d) and the rapid induction of core cold-responsive TFs, such as *CBF4* (Fig. 2e), which collectively function as a critical 'early warning system'. This chromatin remodeling preceded and likely orchestrated subsequent transcriptomic shifts, ensuring the timely activation of downstream pathways in a species that cannot rely on developmental dormancy for cold survival.

The biological significance of this rapid response was further substantiated by shoot apex growth phenotypes following recovery (Fig. 6c, d). Even a transient 0.5 h cold exposure—sufficient to trigger the initial wave of chromatin remodeling—led to a marked inhibition of shoot apex elongation; more prolonged 24-h cold stress resulted in sustained growth suppression. This phenotypic outcome establishes a direct link between early epigenetic hypersensitivity and physiological consequences: cold-induced rapid chromatin and transcriptional reprogramming creates a regulatory conflict between growth-promoting signals and stress adaptation. In this growth–stress trade-off, the prioritization of survival over vegetative growth redirects cellular resources to enhance resilience—a strategy precisely reflected by the temporal hierarchy of regulation defined in this study (Fig. 7).

Cold stress has been reported to activate Ca^2+^ waves and trigger protein post-translational modification^[71]^, while inducing pronounced metabolic reprogramming in rice^[72]^. Consistent with these findings, we observed that short-term cold exposure (0.5–2 h) significantly reshaped chromatin accessibility at loci associated with protein phosphorylation and metabolic processes (Figs 1f, 3d). Leveraging a high-resolution time-series design, we resolved the sequential dynamics of this reprogramming (Fig. 7). Within the initial 0.5 h, accessibility changes occurred most rapidly at hormone- and circadian-related genes. By 2 h, metabolic genes exhibited marked increases in accessibility, followed by cell cycle–related genes at 6 h. Prolonged exposure (12−24 h) predominantly affected genes associated with photoperiodic regulation, flowering, and nutrient-acquisition pathways. Interestingly, the chromatin dynamics of auxin-responsive genes were closely associated with the duration of stress, suggesting a regulatory role for auxin signaling in mediating cold tolerance^[73]^.

Our integrative analyses revealed that over 50% of differential chromatin accessibility events were associated with corresponding changes in gene expression (Fig. 4b, c), suggesting a robust and complex regulatory network. We found that the activation of the master regulator *CBF* may be linked to the basal expression of *AGL42* and *ERF/AP2* TFs in *E. grandis* shoot apices (Fig. 5). Dual-luciferase reporter assays validated this regulatory link: four rapid cold-responsive motifs (0.5 h) identified from ATAC-seq exhibited elevated luciferase activity at 4 °C. More importantly, two putative *E. grandis* *AGL42* homologs significantly enhanced *CBF4* promoter activity, directly confirming that AGL42 transcriptionally regulates *CBF4* (Fig. 6a, b). The positive correlation and partial overlap of AGL42- and ERF-associated target regions suggest their potential coordinated regulation of shared chromatin loci during cold-induced *CBF* activation—a hypothesis requiring further direct experimental validation. These findings suggest that constitutively expressed TFs preconfigure the chromatin landscape of stress-responsive genes, enabling rapid transcriptional responses by bypassing the requirement for *de novo* TF synthesis. Functional validation of the *AGL42*–*CBF4* regulatory relationship not only confirmed the reliability of our chromatin and transcriptomic analyses, but also established a direct molecular link between constitutively expressed developmental TFs and rapid cold signaling activation in *E. grandis* shoot apices. These results further support the model that pre-existing chromatin accessibility at AGL42 binding sites in the *CBF4* promoter provides a key molecular basis for the rapid cold response of this non-dormant subtropical species, effectively accelerating the stress signaling transduction.

While this study provides a high-resolution temporal framework for understanding hierarchical cold signaling, further work is required to identify specific chromatin remodelers and define the precise roles of TFs in recalcitrant woody tissues—efforts currently hampered by the lack of stable *E. grandis* transformation systems. Nevertheless, these findings establish a fundamental molecular basis for elucidating cold adaptation and resilience in forest trees under increasingly volatile environmental conditions.

## Conclusions

This study provides a systematic characterization of time-resolved chromatin accessibility and transcriptional dynamics in *Eucalyptus grandis* shoot apices under cold stress, unraveling the adaptive mechanisms of this non-dormant subtropical perennial. We demonstrated that cold stress triggered a rapid hypersensitive response governed by a hierarchical regulatory architecture, in which over 50% of dynamic chromatin accessibility regions correlated with differential gene expression.

Crucially, we identified a pivotal regulatory relationship wherein the transcription factor AGL42 directly activated the core cold-responsive gene *CBF4*. The presence of pre-existing and rapidly accessible chromatin at the *CBF4* promoter underpinned this near-instantaneous response, which was coupled with a growth–stress trade-off as a central adaptive strategy.

This work addresses a critical gap in the epigenetic regulation of cold tolerance in woody perennials and provides a high-resolution multi-omics resource for the forest biotechnology community. The identified AGL42–CBF4 regulatory relationship offers novel targets for precision genetic improvement, while our methodological framework advances the application of ATAC-seq in recalcitrant woody tissues. Future research will focus on the functional validation of these regulatory relationships and the elucidation of auxin-mediated growth–stress trade-offs to inform targeted breeding. Collectively, this study establishes a fundamental molecular framework for cold adaptation in non-dormant subtropical species, providing a roadmap for the sustainable cultivation and genetic enhancement of *Eucalyptus* in cold-prone regions.

## SUPPLEMENTARY DATA

Supplementary data to this article can be found online.

## Data Availability

The raw reads from RNA-seq and ATAC-seq data that support the findings of this study have been deposited at the China National GeneBank DataBase (CNGBdb) with the Project ID: CNP0008637.
