# Supplementary material for: Chromatin accessibility dynamics and a hierarchical transcriptional regulatory network for shoot apex cold stress in Eucalyptus grandis
Source: For Res (Fayettev). 2026 Apr 13;6:e012. doi: 10.48130/forres-0026-0011 (PMC13195434; doi:10.48130/forres-0026-0011)
Supplement: Supplementary file 1 — Supplementary data to this article can be found online. [file FR-2026-6-0011-S1.zip › 10.48130_forres-0026-0011-Suppl-FigureS6.pdf]

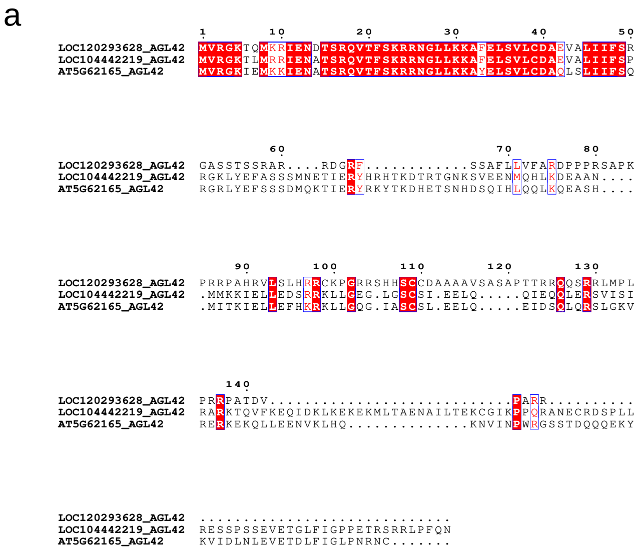

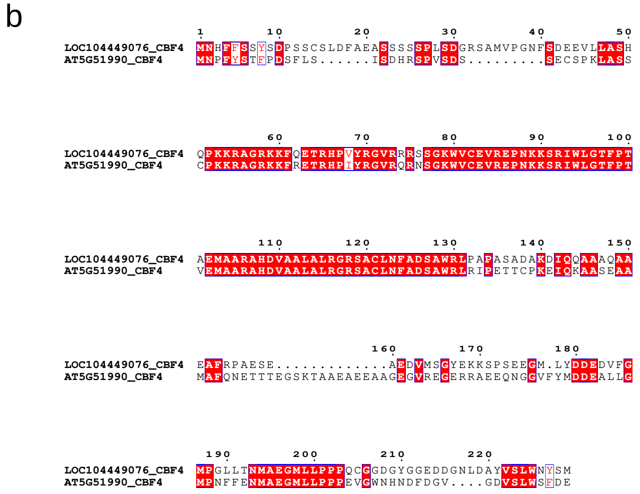

Supplemental Fig. S6 Amino acid sequence alignment of AGL42 (a) and CBF4 (b) between *Eucalyptus grandis* and *Arabidopsis thaliana.*
