# Supplementary figures and images for: Chromatin accessibility dynamics and a hierarchical transcriptional regulatory network for shoot apex cold stress in Eucalyptus grandis
Source: For Res (Fayettev). 2026 Apr 13;6:e012. doi: 10.48130/forres-0026-0011 (PMC13195434; doi:10.48130/forres-0026-0011)

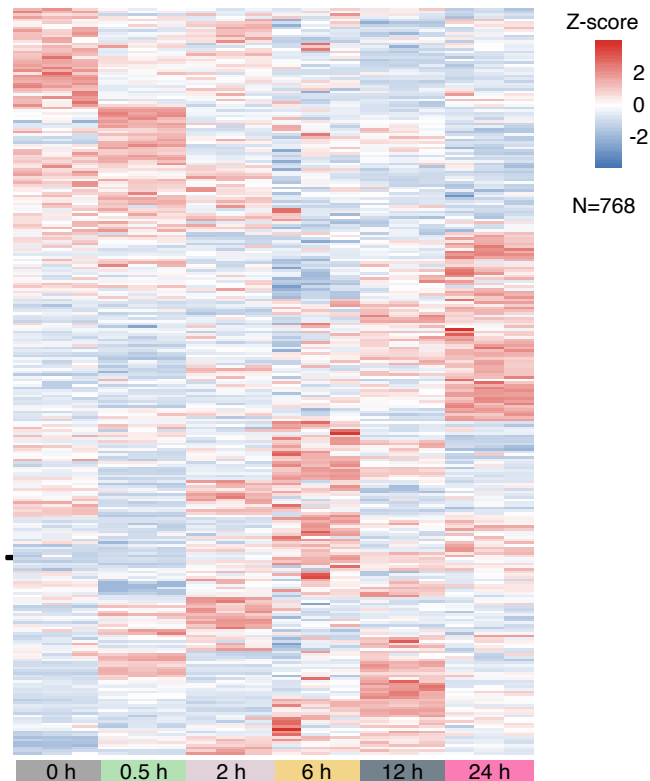

**Supplemental Fig. S2 Expression dynamics of DEGs with dACR feature. N=768.**

Supplement: Supplementary file 1 — Supplementary data to this article can be found online. [file FR-2026-6-0011-S1.zip › 10.48130_forres-0026-0011-Suppl-FigureS2.pdf]

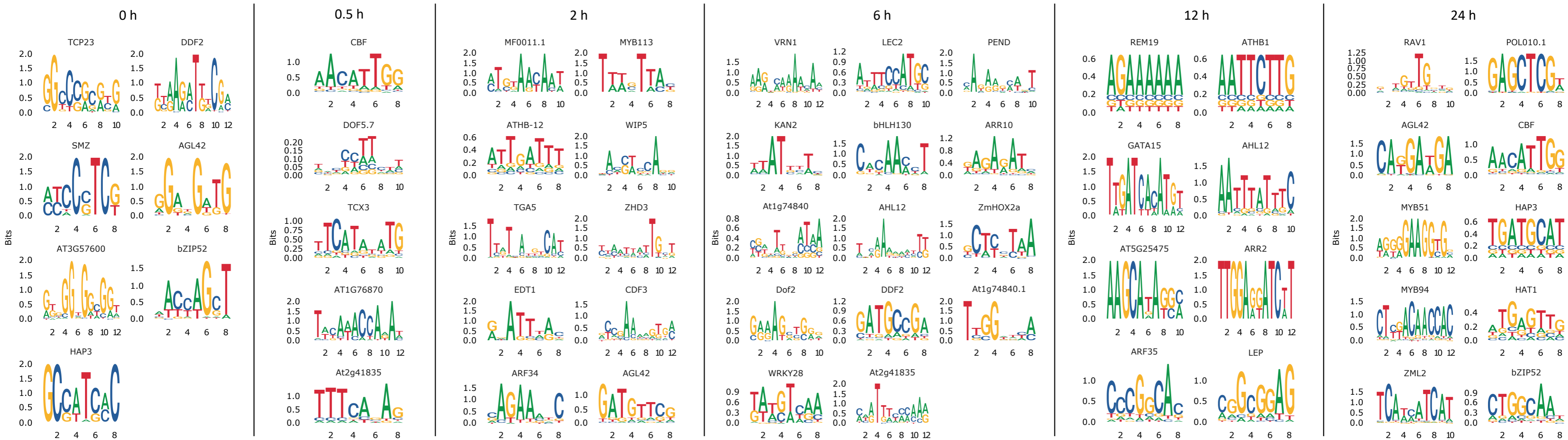

Supplemental Fig. S4 Seqlogo of motifs identified by Homer suit.

Supplement: Supplementary file 1 — Supplementary data to this article can be found online. [file FR-2026-6-0011-S1.zip › 10.48130_forres-0026-0011-Suppl-FigureS4.pdf]

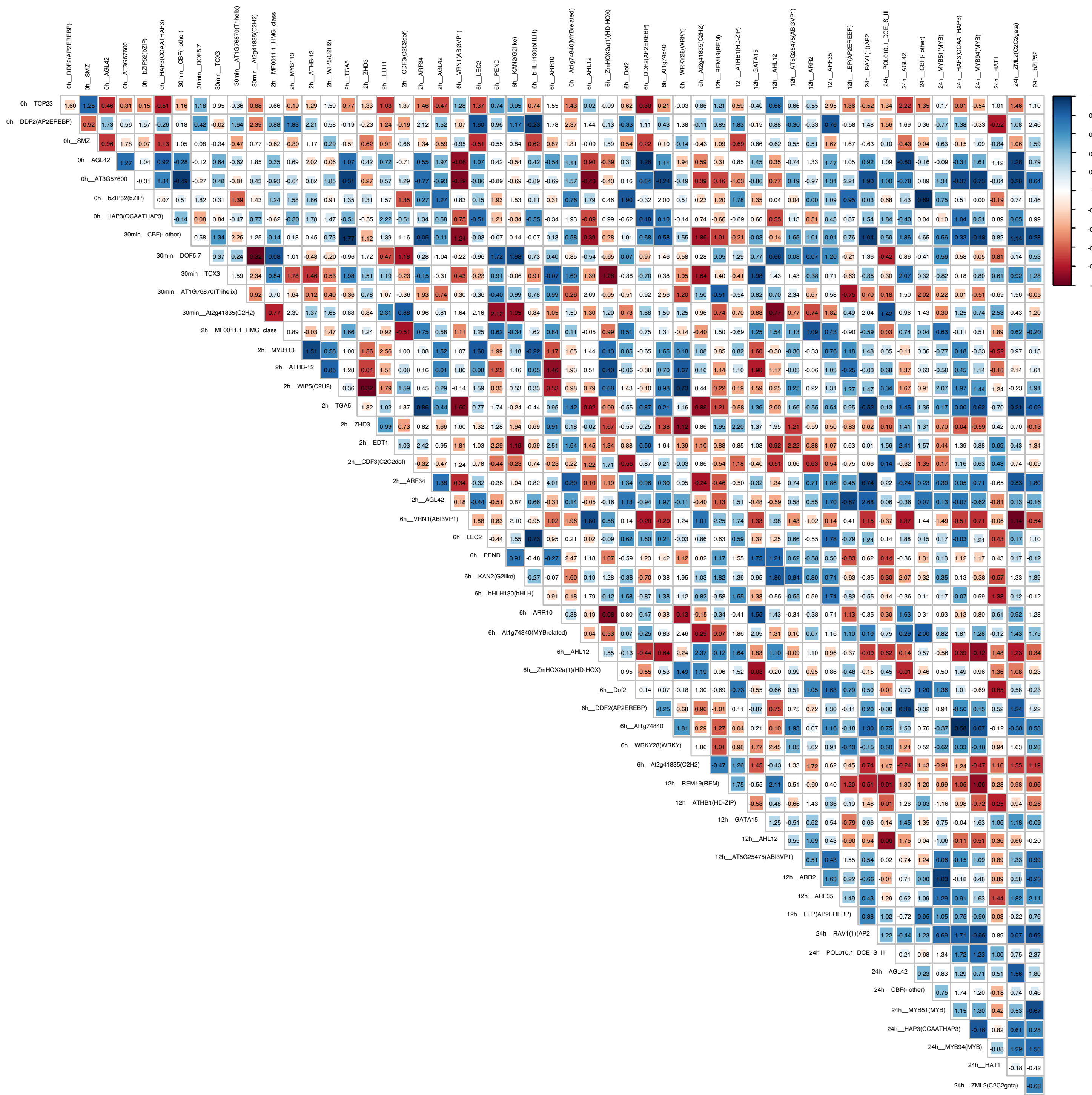

Supplemental Fig. S5 Chromatin-mediated hierarchical gene regulatory networks.

Supplement: Supplementary file 1 — Supplementary data to this article can be found online. [file FR-2026-6-0011-S1.zip › 10.48130_forres-0026-0011-Suppl-FigureS5.pdf]
